# Supplementary material for: TBC1D15 functions as an Arl4D GAP and promotes the mitochondrial translocation of Arl4D for organelle homeostasis
Source: J Cell Sci. 2026 Mar 12;139(5):jcs264304. doi: 10.1242/jcs.264304 (PMC13035273; doi:10.1242/jcs.264304)
Supplement: Supplementary information [file joces-139-264304-s1.pdf]

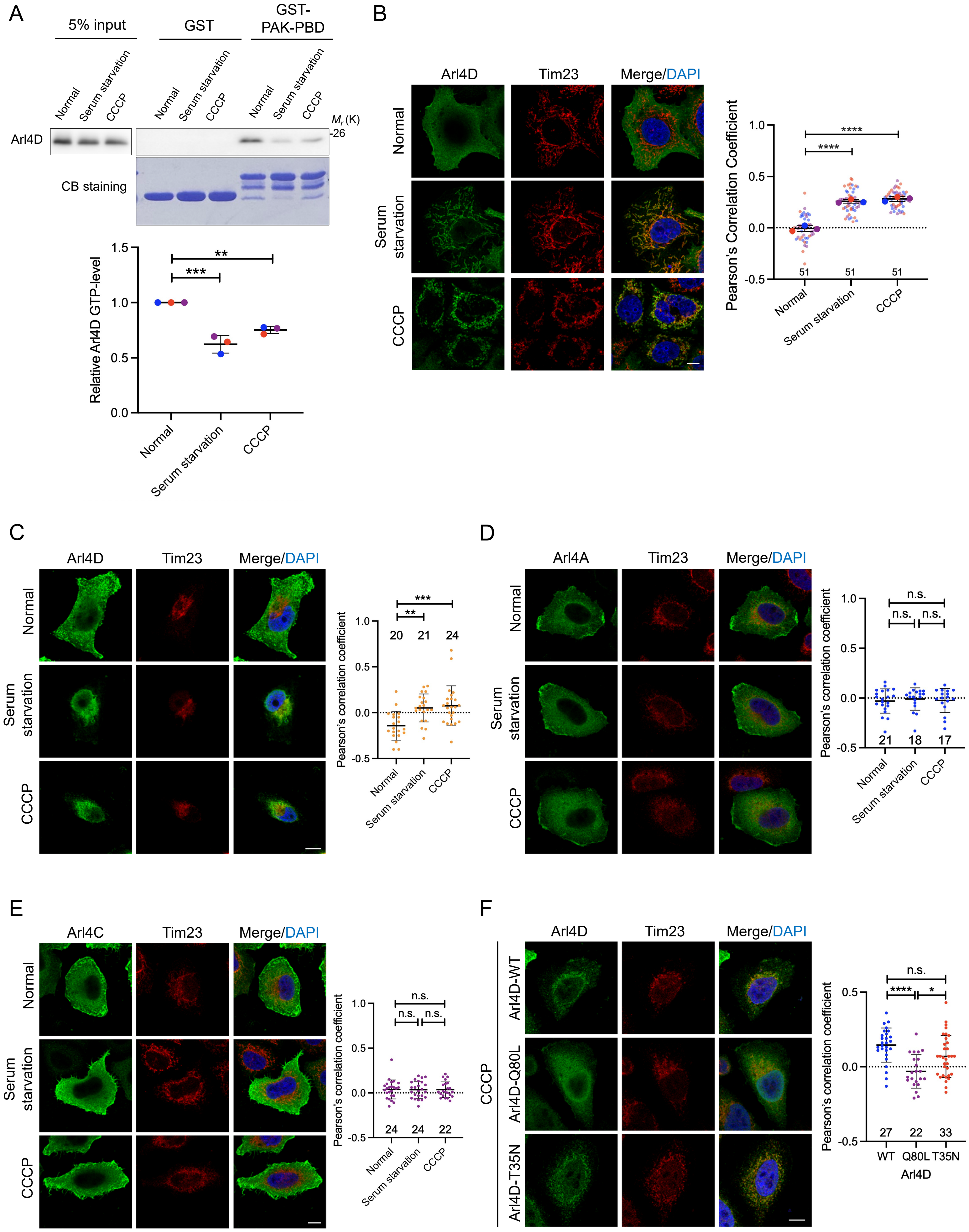

Journal of Cell Science • Supplementary information

Fig. S1. Both serum starvation and CCCP treatment induce translocation and inactivation of Arl4D. (A) HeLa cells were either incubated in normal or serum-free medium for 24 hours or incubated in 20 μM CCCP for 2 hours before harvesting the lysate. Activated Arl4D was pulled down by GST-Pak1-PBD and quantified by densitometric methods. Data are presented as mean±SD (n=3) and analyzed by one-way ANOVA with Turkey's post hoc multiple comparison test. (B, C) HeLa cells (B) or MDA-MB-231 cells (C) were transfected with Arl4D and incubated with either normal or serum-free medium for 24 hours or normal medium with 20 μM CCCP for 2 hours. (D, E) HeLa cells transfected with the Arl4A (D) or Arl4C (E) plasmid were incubated in either normal or serum-free medium for 24 hours or normal medium with 20 μM CCCP for 2 hours. Cells were then fixed and stained for anti-Arl4A/C/D (green), anti-Tim23 (red) and DAPI (blue). (F) HeLa cells transfected with Arl4D-WT/Q80L/T35N were incubated in 20 μM CCCP for 2 hours before fixation and staining for anti-Arl4D (green), anti-Tim23 (red) and DAPI (blue). Quantification of colocalization of Arl4D and Tim23 is represented by Pearson's correlation coefficient. Data are presented as mean±SD (*p*-value was determined by one-way ANOVA). Scale bar, 10 μm.

Journal of Cell Science • Supplementary information

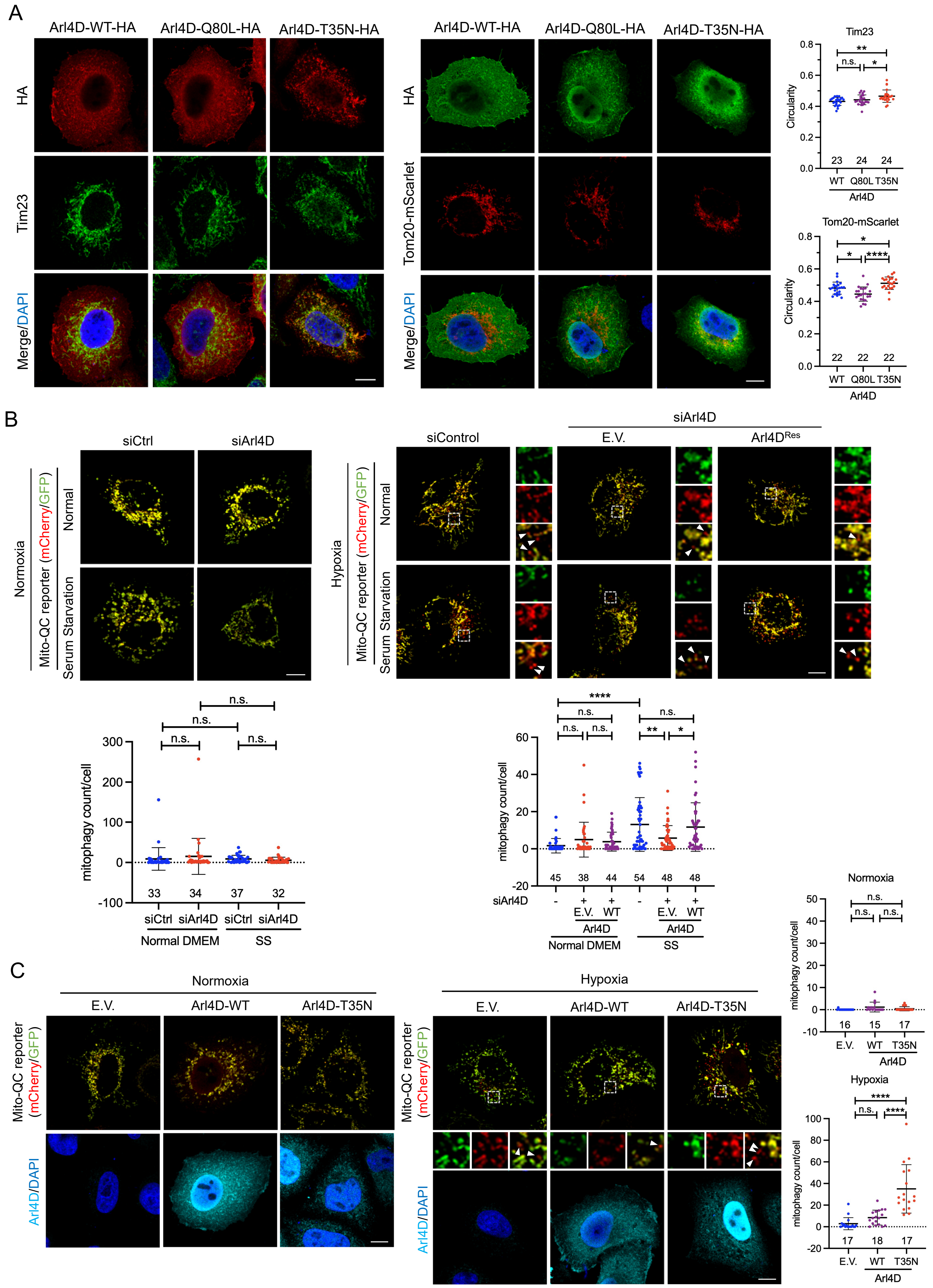

Journal of Cell Science • Supplementary information

Fig. S2. Arl4D regulates mitochondrial morphology and homeostasis. (A) HeLa cells were transfected with HA-tagged Arl4D WT/Q80L/T35N and stained for anti-HA (red), anti-Tim23 (green) and DAPI (blue) in left panel. In right panel, cells were additionally co-transfected with Tom20-mScarlet before staining for anti-HA (green) and DAPI (blue). Mitochondria fragmentation was assessed by quantifying the circularity of Tim23 or Tom20-mScarlet fluorescence signals by FIJI (ImageJ2) software. At least 20 cells were quantified for every group. (B) HeLa cells incubated with indicated siRNAs were further transfected with mito-QC reporter with or without Arl4D rescue before cultured in normal or serum-free medium under normoxia or hypoxia (0.5% O_2) conditions for 24 hours. Mitophagy counts were presented as mean±SD. Insets show red fluorescence signals indicated with arrowheads that are considered as actual mitophagy counts. (C) HeLa cells co-transfected with Arl4D-WT/T35N and mito-QC reporter incubated under normoxia or hypoxia conditions were stained for anti-Arl4D (cyan) and DAPI (blue). Mitophagy counts were presented as in (B). *p*-values were analyzed by one-way ANOVA with Turkey's post hoc multiple comparison test. SS, serum starvation. *P<0.05, **P<0.01, ***P<0.001, ****P<0.0001. Scale bar, 10 μm.

Journal of Cell Science • Supplementary information

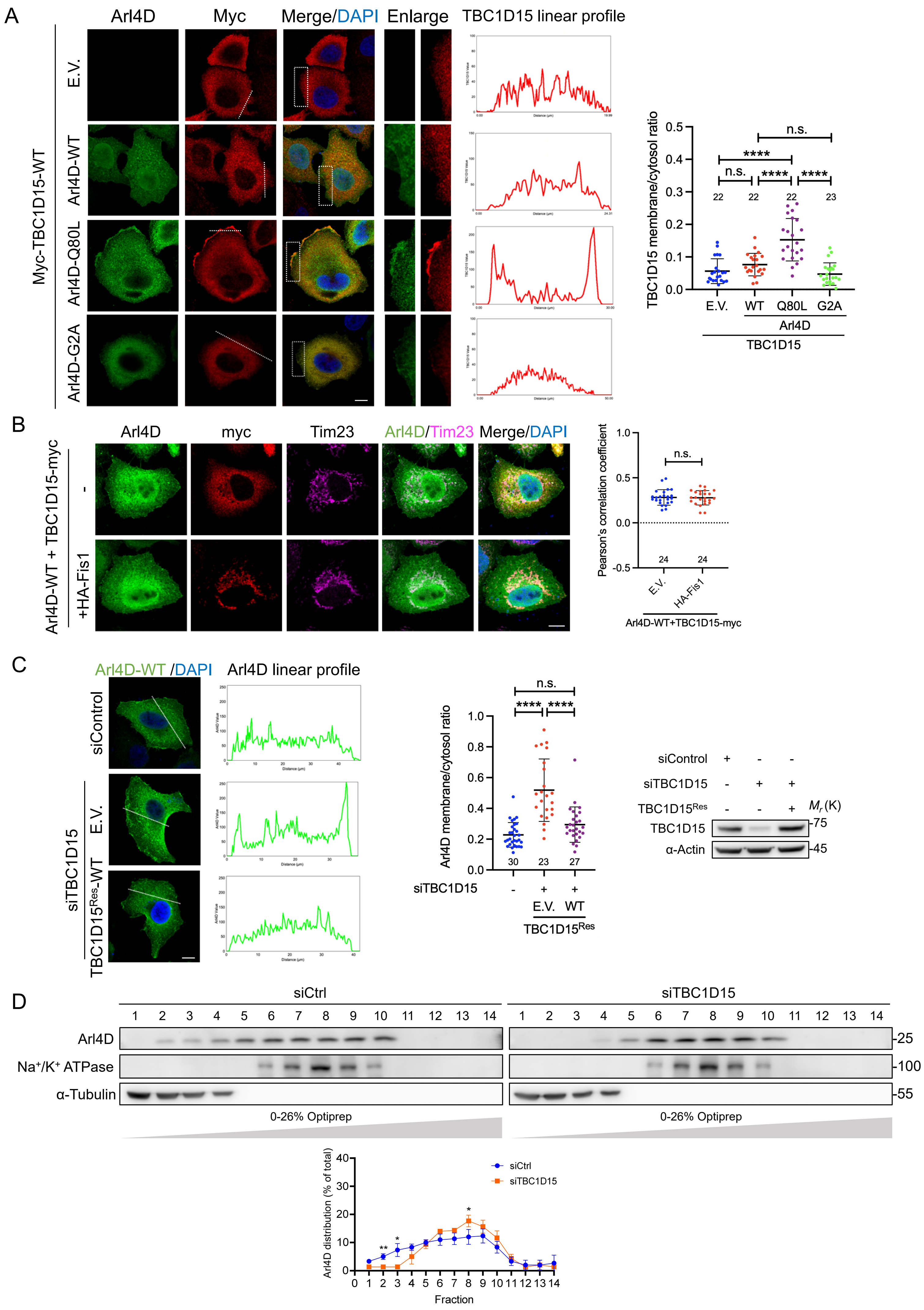

Journal of Cell Science • Supplementary information

Fig. S3. TBC1D15 regulates the localization of Arl4D at the plasma membrane. (A) HeLa cells co-expressing Myc-TBC1D15 and Arl4D WT/Q80L/G2A were stained for anti-Arl4D (green), anti-Myc (red) and DAPI (blue). (B) HeLa cells co-transfected with Myc-TBC1D15 and Arl4D WT with or without HA-Fis1 were stained for anti-Arl4D (green), Myc (red), Tim23 (magenta) and DAPI (blue). (C) HeLa cells transfected with siControl or siTBC1D15 and subsequently expressed with Arl4D plasmid with or without TBC1D15 rescue were stained for anti-Arl4D (green) and DAPI (blue). The efficiency of TBC1D15 knockdown was checked by immunoblotting. (D) Lysates from HeLa cells treated with indicated siRNAs were subjected to subcellular fractionation to assess plasma membrane translocation. Quantification shows the percentage of total protein in each fraction.(A)(B)(C) Quantitative ratios between plasma membrane and cytosol of Arl4D, or the colocalization of Arl4D (represented by Pearson's correlation coefficient), are presented as mean±SD ($n \geq 20$ cells per group). P-value was obtained by one way ANOVA with Turkey's post hoc multiple comparison test. ***P<0.001, ****P<0.0001. Scale bar, 10 µm.

Journal of Cell Science • Supplementary information

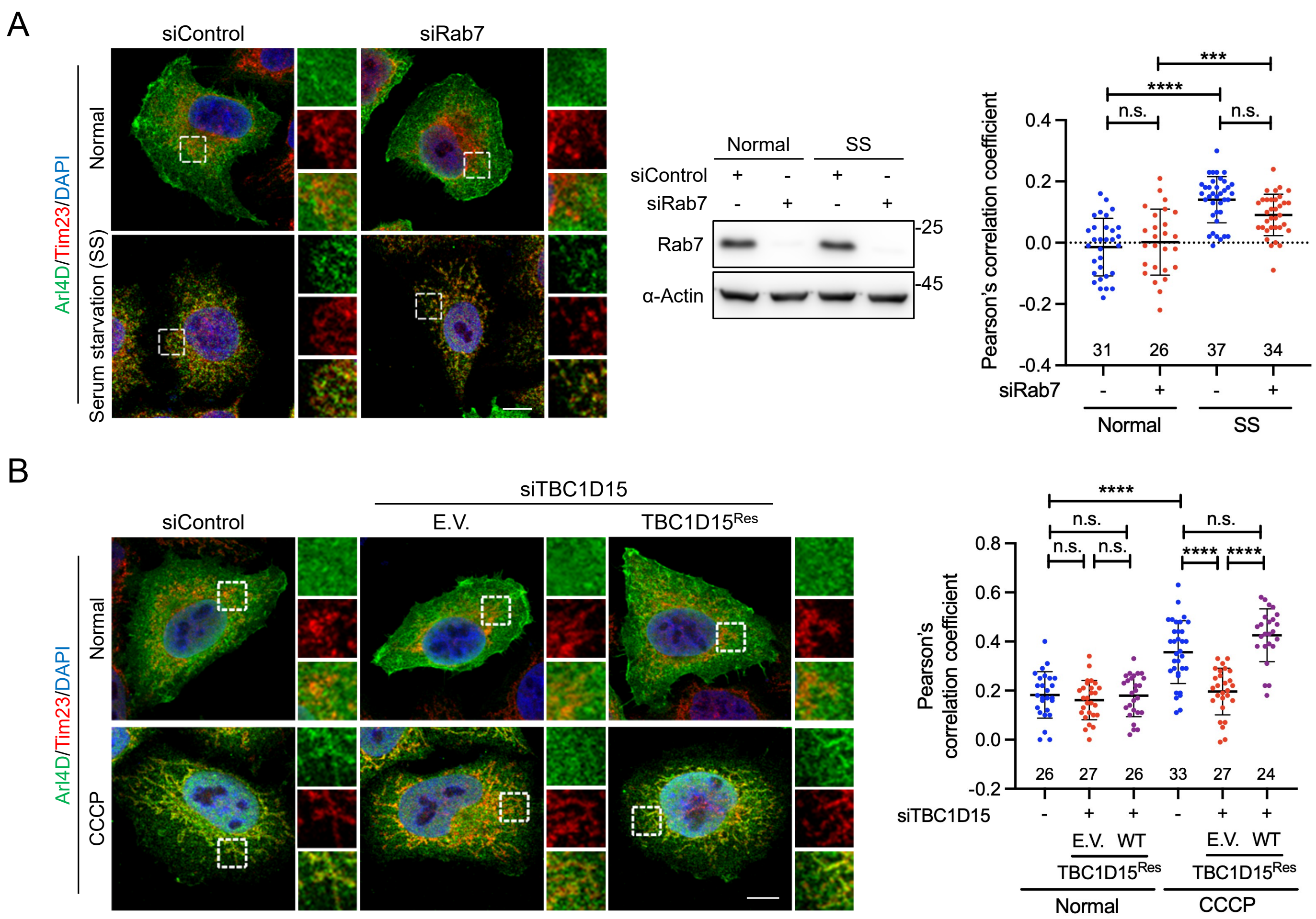

Fig. S4. TBC1D15 depletion inhibits translocation of Arl4D to mitochondria. (A) HeLa cells were treated with control siRNA (siControl) or Rab7 siRNA (siRab7) prior to transfection with Arl4D and incubation in either complete or serum-free DMEM for 24 hours. (B) HeLa cells treated with either siControl or siTBC1D15 were transfected with the Arl4D plasmid before incubation with normal DMEM or DMEM with 20 μM CCCP for 2 hours. Cells were then fixed and stained for anti-Arl4D (green), anti-Tim23 (red) and DAPI (blue). Quantification of colocalization of Arl4D and Tim23 is represented by Pearson's correlation coefficient. Data are presented as mean±SD (*p*-value was determined by one-way ANOVA). Scale bar, 10 μm.

Journal of Cell Science • Supplementary information

Figure 1A

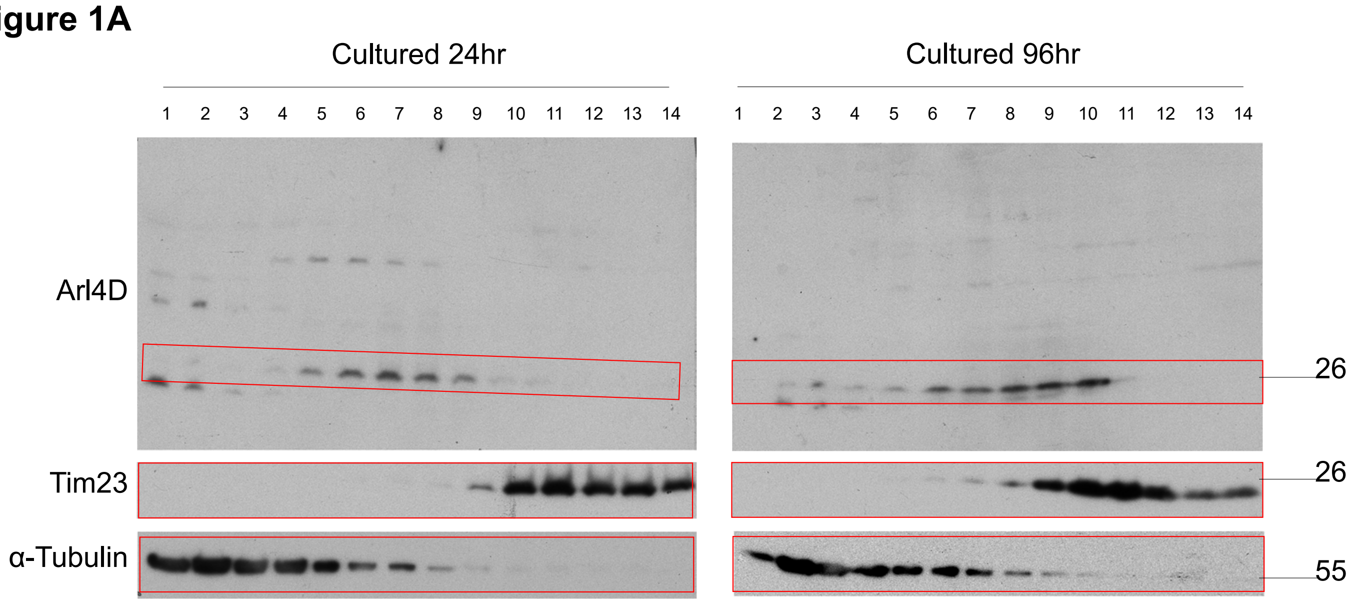

Figure 1C

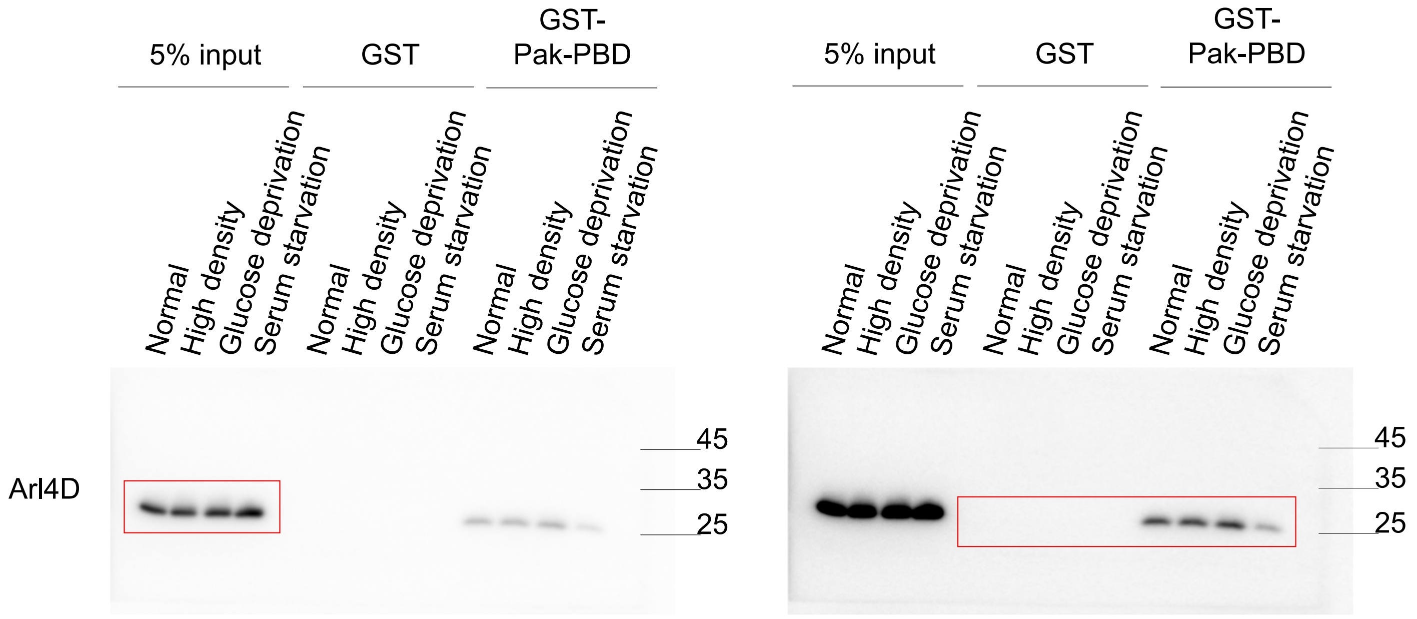

Figure 1E

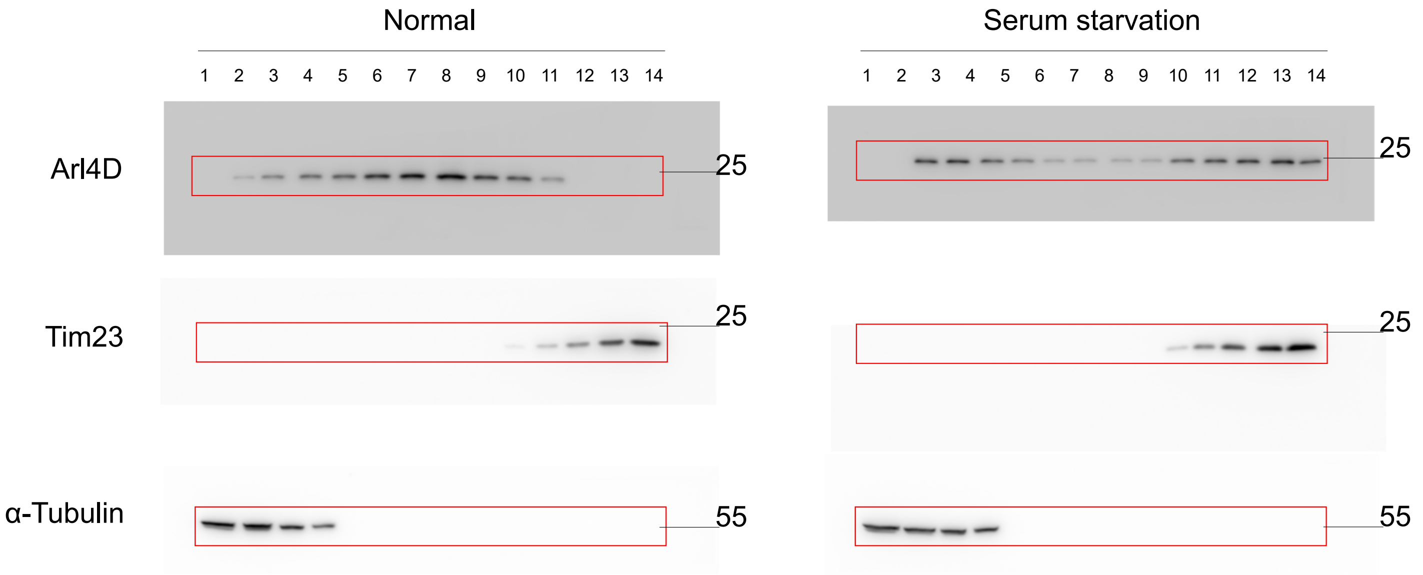

Figure 2C

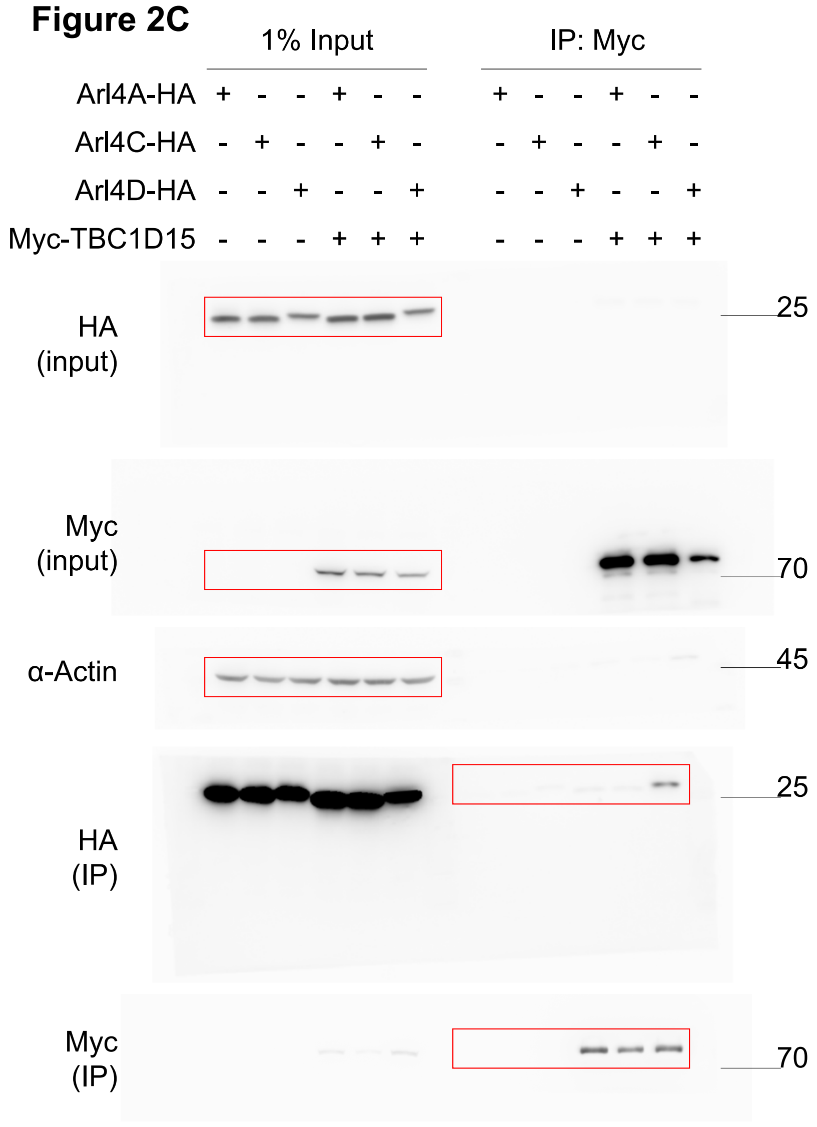

Figure 2E

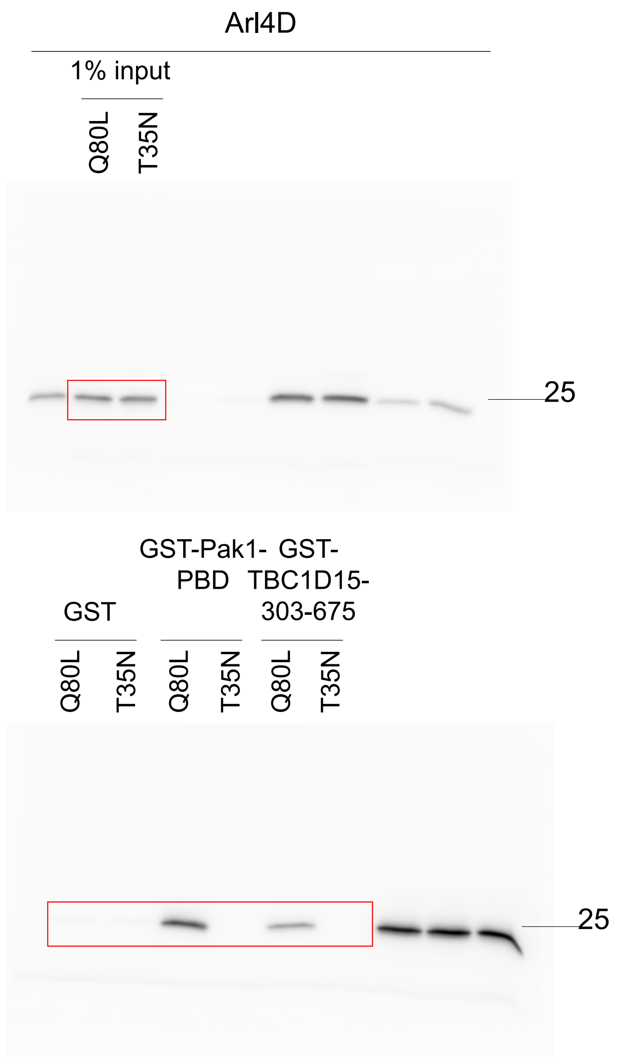

Figure 2D

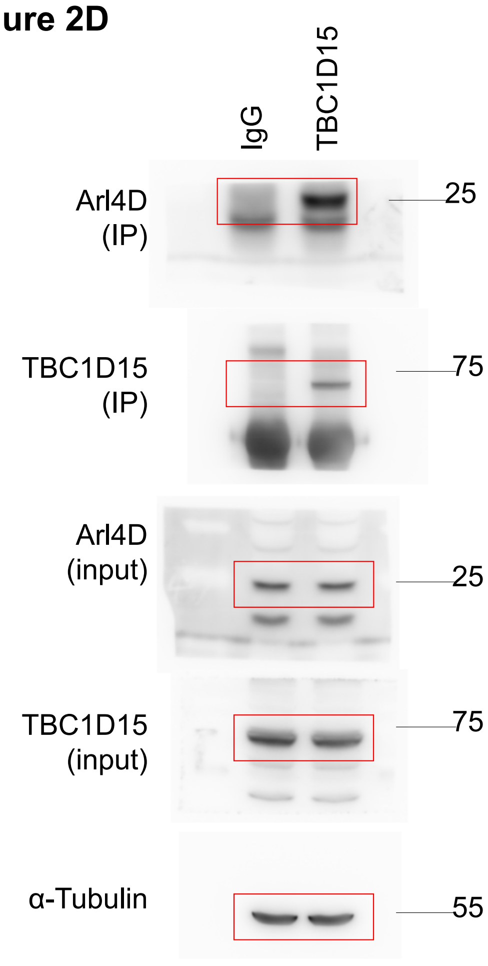

Figure 2F

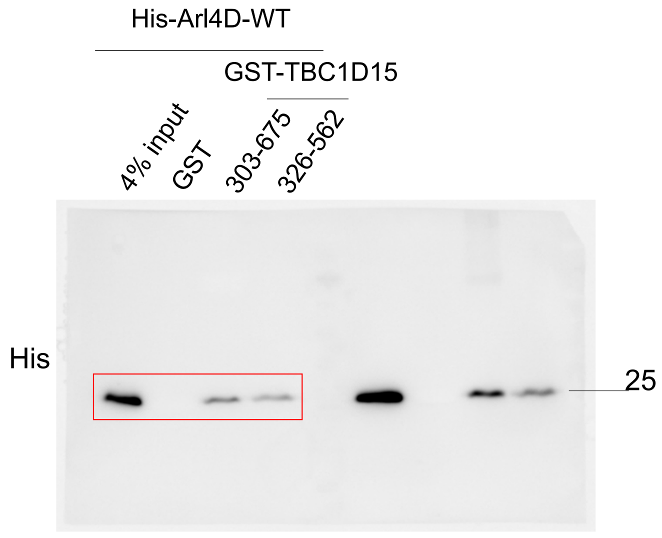

Journal of Cell Science • Supplementary information

Figure 2G

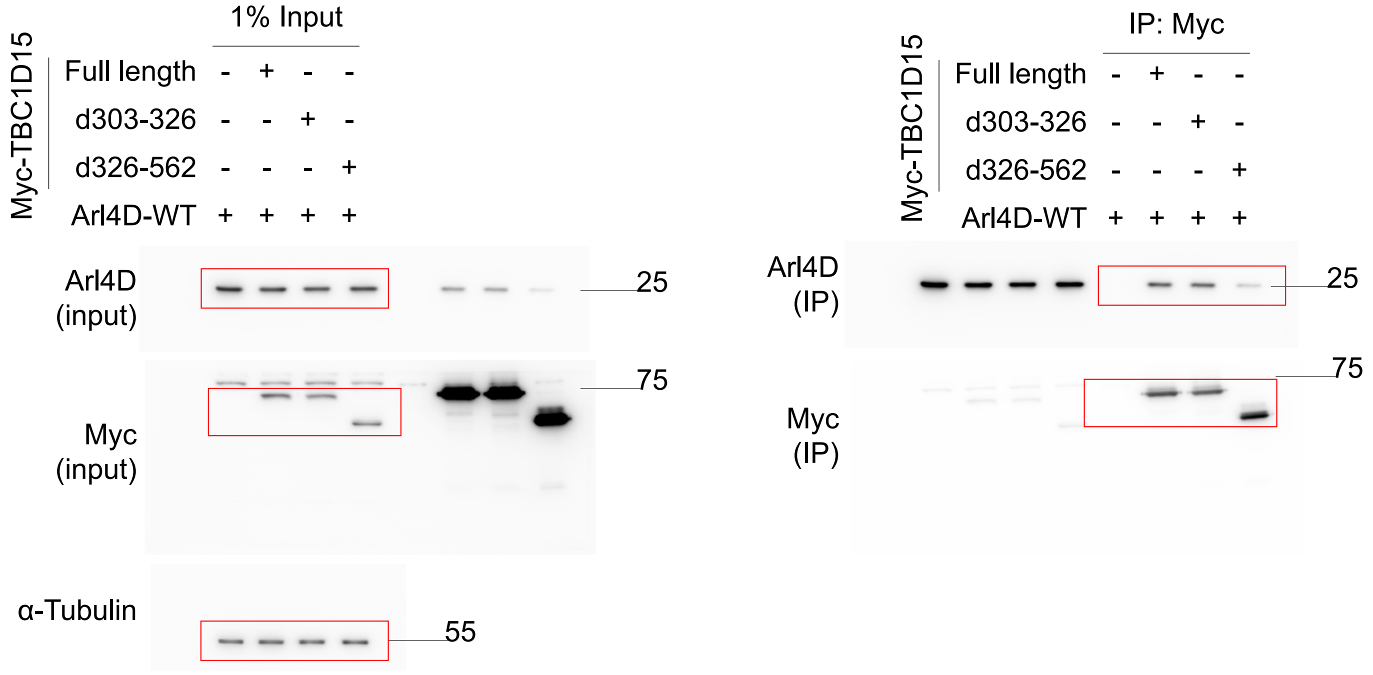

Figure 3C

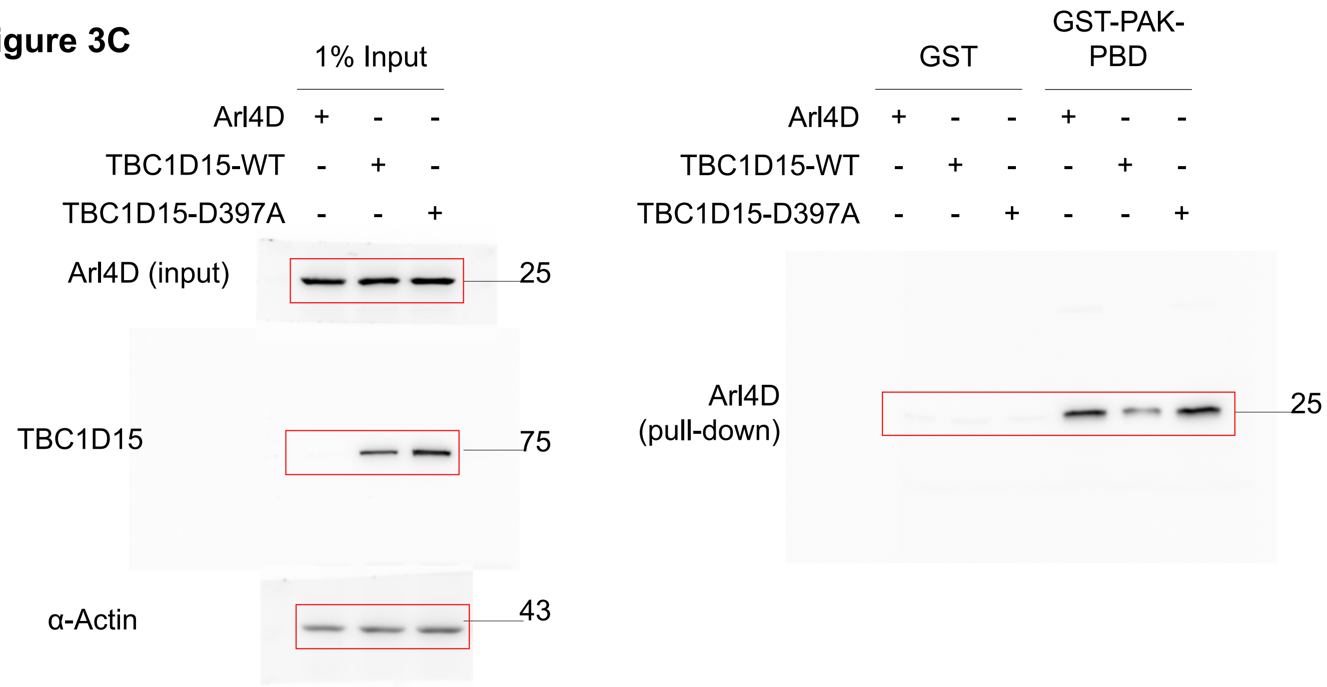

Figure 3D

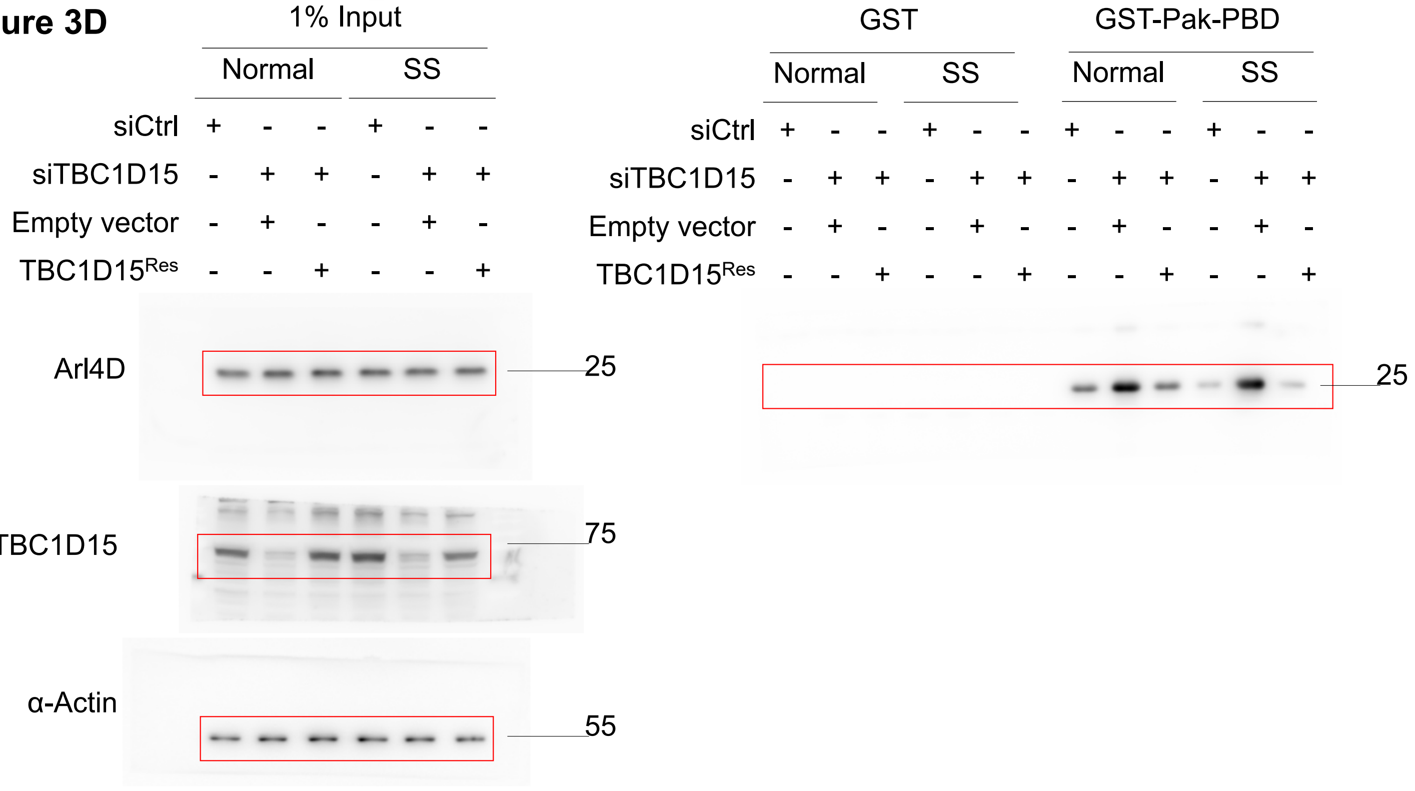

Journal of Cell Science • Supplementary information

Figure 4A

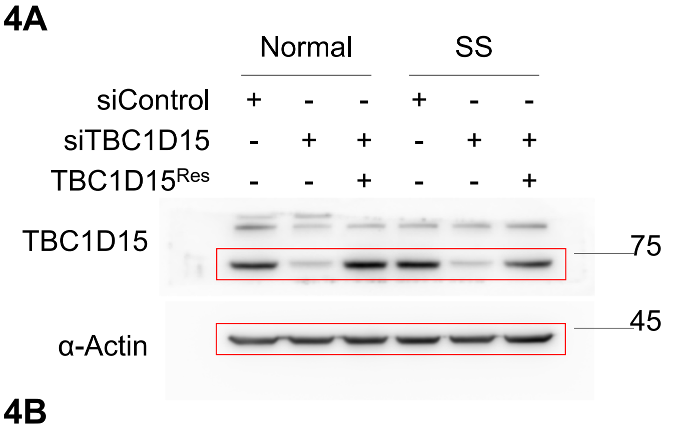

Figure 4B

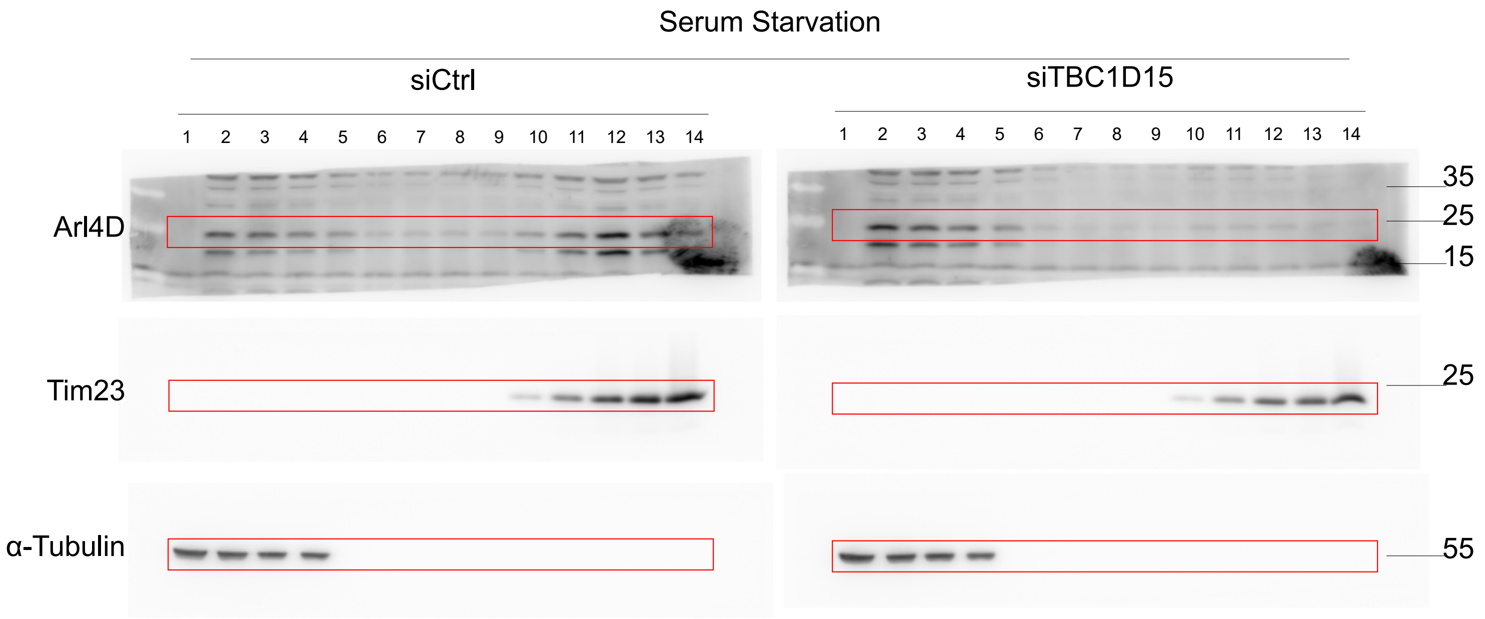

Figure 4C

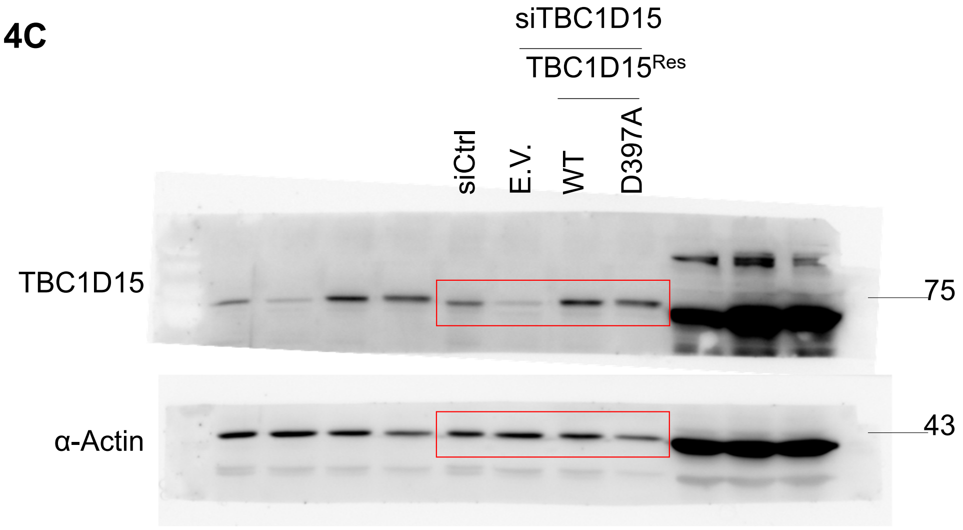

Figure S1A

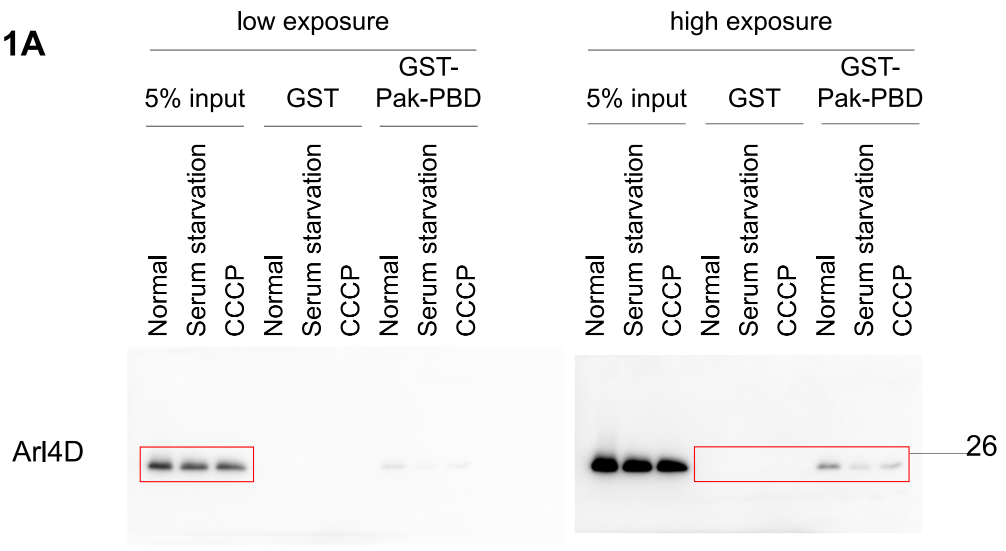

Journal of Cell Science • Supplementary information

Figure S3B

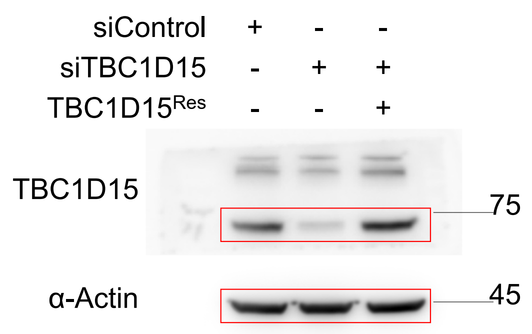

Figure S3C

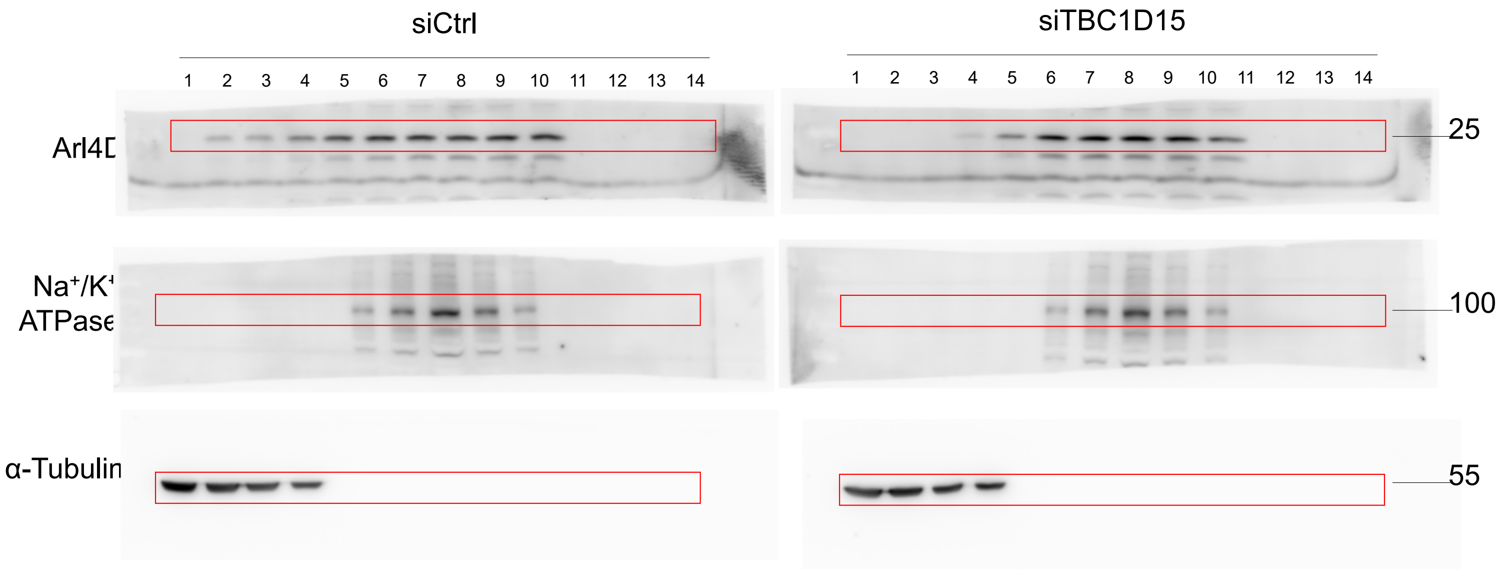

Figure S4A

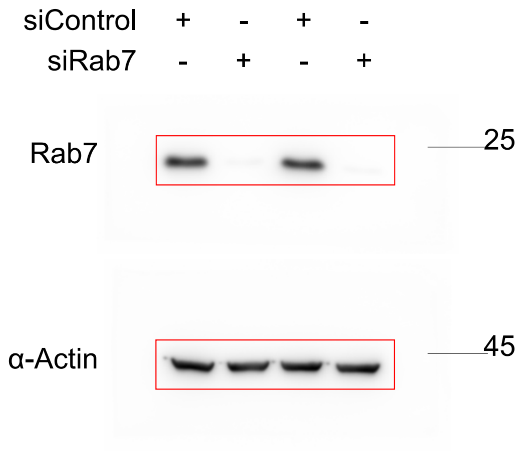

Fig. S5. Blot Transparency. Show the original image of Western Blotting.

Journal of Cell Science • Supplementary information
